# The relationship between autophagy and apoptosis during pseudorabies virus infection

**DOI:** 10.3389/fvets.2022.1064433

**Published:** 2022-12-20

**Authors:** Mingxia Sun, Linlin Hou, Huan Song, Chuang Lyu, Yan-dong Tang, Lei Qin, Yonggang Liu, Shujie Wang, Fandan Meng, Xuehui Cai

**Affiliations:** ^1^State Key Laboratory of Veterinary Biotechnology, Harbin Veterinary Research Institute of Chinese Academy of Agricultural Sciences, Harbin, Heilongjiang, China; ^2^Heilongjiang Provincial Key Laboratory of Veterinary Immunology, Harbin Veterinary Research Institute, Chinese Academy of Agricultural Sciences, Harbin, China; ^3^Laboratory Animal Centre, Qiqihar Medical University, Qiqihar, China

**Keywords:** swine pseudorabies virus, autophagy, apoptosis, ROS, cross-talk

## Abstract

Both autophagy and apoptosis are mechanisms that maintain homeostasis in cells and that play essential roles in viral infections. Previous studies have demonstrated that autophagy and apoptosis pathways occurred with complex relationships in virus-infected cells. However, the regulation between these two processes in Pseudorabies virus (PRV) infection remains unclear. In the present study, we demonstrated that activated autophagy was induced at the early stage of PRV infection and that apoptosis was induced at the late stage of infection. Autophagy induction inhibited apoptosis and decreased viral replication, and autophagy inhibition promoted apoptosis and increased viral replication. We also found that viral infection resulted in an increase in the production of reactive oxygen species (ROS) and activation of apoptosis in autophagy-impaired cells, suggesting that ROS may participate in the cross-talk between autophagy and apoptosis in PRV-infected cells. Our studies provide possible molecular mechanisms for the cross-talk between apoptosis and autophagy induced by PRV infection in porcine cells. This suggests that these two cell death processes should be considered as the same continuum rather than as completely separate processes.

## Introduction

Pseudorabies virus (PRV) is a swine herpesvirus in the *Alphaherpesvirinae* subfamily. Pigs are considered to be the natural reservoir of PRV that occasionally also infects cattle and dogs. PRV infection in pigs may induce Aujeszky's disease (AD) that results in significant economic losses worldwide (1–3). Recently, several cases have been reported implying a potential risk of PRV crossing the species barrier and infecting humans, causing severe diseases such as encephalitis and respiratory dysfunction (4, 5). It has been reported that the first human-originated isolate hSD-1/2019 strain shows a high phylogenetic relationship and etiological characteristics similar to strains prevalent in the pig population (4). Therefore, there is a potential risk for people that have contact with pigs to acquire PRV infection.

PRV infection induces rapid and extensive changes in cultured host cells. PRV is known to induce apoptosis in infected immortalized cells (3, 6). However, the underlying mechanisms are not fully understood. Alphaherpesviruses encode several anti-apoptotic genes to promote the survival in trigeminal ganglion neurons and the establishment of latent infection (7, 8). US3 is one of the most potent and best-characterized anti-apoptosis genes and is the only PRV gene found to have anti-apoptosis activity (9). The US3 protein kinase may block apoptosis by preventing Bad phosphorylation and activating PI3-K/Akt and NF-κB pathways (10, 11). Autophagy is an evolutionarily conserved catabolic process in eukaryotic cells during which lysosomes degrade cellular components, including long-lived proteins and organelles (12–14). Cell death is often triggered by autophagy, but it is not actually mediated by autophagy (15).

In some cases, autophagic membranes or autophagy-relevant proteins may promote the activation of apoptosis or necrosis. Therefore, apoptosis and autophagy are two important cellular processes that not only participate in a wide range of diseases and regulate pathogen infections, but also are highly interconnected in determining the fate of cells. Though the relationship between autophagy and apoptosis has been a focus topic (16–20), it is subject to variation depending on different circumstances. Many studies have demonstrated that the balance between autophagy and apoptosis is regulated by the molecular interactions involving classical apoptosis- and autophagy-associated molecules (16, 21). However, the regulation of autophagy and apoptosis during PRV infection is far from being understood. Thus, our investigation aims to provide clues that help to elucidate the pathogenesis of PRV.

In this paper, we studied the relationship between autophagy and apoptosis in PRV-infected PK-15 cells (porcine kidney epithelial cells) and monitored the appearance of these processes in the course of infection. Autophagy was detected early in infection, whereas apoptosis prevailed at the late stage. Furthermore, PRV-induced apoptosis was affected when cellular autophagy was modified by treatment with chemical inducers or inhibitors. On the other hand, PRV-induced autophagy was increased when apoptosis was inhibited. These findings provide a deeper insight into the virus-host interactions, which may help to develop new antiviral treatments directed to apoptosis and autophagy pathways as novel targets for antiviral therapy.

## Materials and methods

### Cell cultures and viruses

PK-15 cells and Vero cells were cultured in Dulbecco's modified Eagle medium (DMEM) supplemented with 10% (v/v) fetal bovine serum (FBS) at 37°C in 5% CO_2_ atmosphere. The PRV strain HeN1 was isolated and stored in our laboratory. The PRV stock was propagated in PK-15 cells with 2% FBS. After incubation for 3 or 4 days, the culture medium was harvested when the cytopathic effect (CPE) was apparent, and the supernatant was used for virus titration. The titer of the virus was 1.2 × 10^7^ plaque-forming units per ml (PFU/ml).

### Chemicals and antibodies

Rapamycin (R0395), 3-MA (3-Methyladenine, M9281) and Z-VAD-FMK (N-Benzyloxycarbonyl-Val-Ala-Asp (O-Me) fluoromethyl ketone, V116) were purchased from Sigma-Aldrich (USA). Anti-β-actin (ACTB, A5316), anti-ATG5 (A0731) antibody and polyclonal anti-LC3 (Microtubule-associated protein 1A/1B-light chain 3, L7543) antibody were purchased from Sigma-Aldrich (USA); anti-cleaved caspase3 antibody (AC033) was purchased from Beyotime (China, Shanghai); anti-gE antibody was produced from immunized mice. NAC (N-acetyl-L-cysteine, S0077) and a Reactive Oxygen Species Assay Kit (S30033) were purchased from Beyotime. A Cell Death Detection ELISA PLUS Kit (11774425001) was purchased from Roche (USA).

### Cell culture and virus infection

Cells were infected with PRV at an MOI of 1 or 10, as indicated. After incubation for 1 h at 37°C, the unbound virus was removed by washing the cells three times with PBS. The cells were then cultured in DMEM supplemented with 2% FBS for the indicated times.

### Western blot analysis

Western blot (WB) was performed as previously described (22). Briefly, the cells were collected and incubated in RIPA buffer containing a protease inhibitor cocktail (Roche) and 0.1 mM PMSF for 2 h. The protein concentration of the cell lysate supernatants was determined by BCA assay (Thermo). Equal amounts of protein were diluted in 5 × SDS-PAGE loading buffer and separated on SDS-PAGE gels. The proteins were blotted on polyvinylidene fluoride (PVDF) membranes (Millipore, USA), then blocked with 5% non-fat dry milk in PBST for 2 h and incubated with primary antibodies at 4°C overnight. The membranes were incubated with secondary antibodies in a blocking solution. The reactive bands were detected by using the enhanced chemiluminescence system (ECL).

### Flow cytometry

Flow cytometric analysis was performed as previously described (23). PK-15 cells were infected or mock-infected with PRV, followed by the addition of a chemical inducer or inhibitor. The cells were harvested after having been washed in cold PBS, and stained with FITC-labeled annexin V and PI (556547, BD) at room temperature for 15 min and analyzed by flow cytometer (BD Canto II) within half an hour. The cells in the second quadrant (annexin V-positive/PI-negative) were considered early apoptotic cells, and the cells in the fourth quadrant (annexin V-positive/PI-positive) were considered late apoptotic cells. Both of them were considered to be apoptotic cells.

### Plaque formation assay

Ten-fold dilutions of the virus-containing culture supernatants were added to 6-well plates with a confluent monolayer of Vero cells. The plates were then incubated at 37°C for 2 h, with gentle agitation at 30 min intervals. The excess virus was washed away with PBS. Subsequently, 2% low-melting-point agarose and 2 × DMEM containing 2% FBS were mixed and added to each well as an overlay medium. The cells were incubated at 37°C with 5% CO_2_ for 3–4 days, then stained with 0.5% crystal violet, and the plaques were counted.

### Transfection and RNA interference

PK-15 cells were grown to 80% confluences in 6-well plates and transiently transfected with the indicated shRNAs (Supplementary Table 1) by using the transfection reagent (Roche) according to the manufacturer's instructions. Western blot was used to detect the silencing efficiency of the shRNAs.

### Assay of intracellular reactive oxygen species (ROS)

ROS was measured using the non-fluorescent probe 2′,7′-dichlorofluorescein diacetate (DCFH-DA) as previously described (24). DCFH-DA is deacetylated by esterase to form non-fluorescent DCFH after diffusing into cells passively. ROS were produced when cells were under oxygen stress, and the fluorescent product DCF was formed from DCFH. PK-15 cells were transfected with shATG5 and shNC and infected with PRV 24 h later. Fifty millimeter NAC was added at the infection time to eliminate ROS. Ten micrometer DCFH-DA was added to cultures and incubated for 20 min at 37°C. After incubation, cells were washed in cold PBS and analyzed by flow cytometry. The fluorescent intensity was calculated by Image J software.

### Statistical and image analysis

All dates were analyzed in SPSS. One-way ANOVA was used to evaluate the difference among one variable treatment. Two-way ANOVA was used to evaluate the difference among treatment groups. The data is expressed as the mean ± standard deviation (S.D.). *P*-value < 0.05 was considered statistically significant. The intensities of the immune bands were analyzed using Image J software (HIN).

## Results

### PRV-induced caspase-dependent apoptosis in infected PK-15 cells

**Figure 1:**
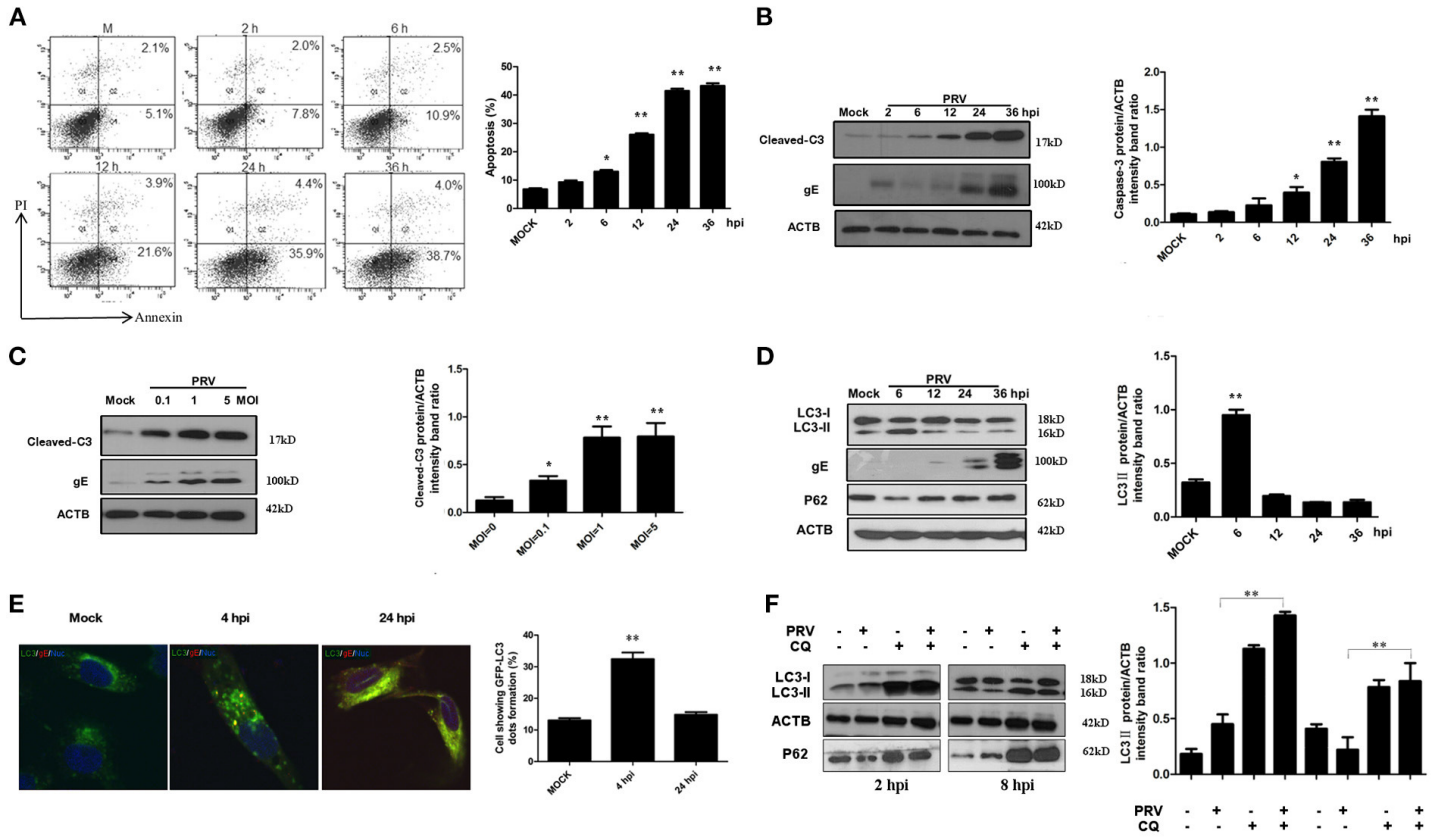
PRV induces caspase-dependent apoptosis and autophagy in different stages of infection. **(A)** PK-15 cells were infected with PRV at the MOI of 1 at different times, and apoptotic cells were analyzed by flow cytometry. The percentage of apoptotic cells was represented as the histogram. **(B)** PK-15 cells infected with the PRV virus at the MOI of 1 and lysed at indicated time points, and the cell extracts were analyzed by Western blot. The ratios of intensity of cleaved caspase-3 to ACTB were calculated. **(C)** PK-15 cells were infected with PRV at different MOIs for 24 h. Then the cells were harvested and analyzed by Western blot. The ratios of intensity of cleaved caspase-3 to ACTB were calculated. **(D)** The cells were infected with PRV at the MOI of 0.1. Cells were lysed at different time points and analyzed by Western blot. The ratio of the intensity of LC3-II to ACTB was calculated to represent the autophagic level. **(E)** PK-15 cells were infected with PRV following transfected with GFP-LC3 for 24 h and then detected by confocal microscopy. The PRV gE protein was stained in red. The cells showing GFP-LC3 dot formation and cells expressing GFP were counted, and at least 50 cells were included in each group. The results were represented as the histogram. **(F)** PK-15 cells were pre-treated with CQ for 4 h, followed by PRV infection at an MOI of 1. Cell samples were harvested at 2 and 8 hpi, and analyzed by Western blot. The ratio of the intensity of LC3-II to ACTB was calculated to represent the autophagic level. All the dates are representative of three independent experiments. The data represent the mean ± SD, and analyzed by two-way ANOVA, *P < 0.05; **P < 0.01.

To detect the apoptotic effects of PRV, we used the PK-15 cell line, which is highly susceptible to infection. PK-15 cells were infected by PRV at an MOI of 1, and then the percentage of apoptotic cells among infected PK-15 cells was determined by flow cytometric analysis at different time points. The results shown in Figure 1A demonstrate that PRV infection causes apoptosis at 12 h post-infection (hpi) and later (*P* < 0.01), but not at earlier time points. The percentage of apoptotic cells shown in the histogram (Figure 1A) ranged between 25% and 40%. The amount of cleaved caspase-3 and PRV gE protein was detected by Western blot and the ratios of intensity of cleaved caspase-3 to ACTB were determined. Consistent with the results of flow cytometry, PRV-induced cleaved caspase-3 was detectable beginning at 12 hpi, and significant cleavage was found at 24 and 36 hpi (*P* < 0.01) (Figure 1B). In order to analyze the effect of the infectious dose on virus-induced apoptosis, PK-15 cells were infected by PRV at 0.1, 1 and 5 MOI for 24 h. The levels of cleaved caspase-3 and PRV gE protein were visualized by Western blot, and the ratios of intensity of cleaved caspase-3 to ACTB were determined. The cleavage of caspase-3 was increased along with the infection dosage in a dose-dependent manner, and an MOI of PRV as low as 0.1 can induce significant apoptosis (*P* < 0.05) (Figure 1C). Taken together, we confirmed that PRV is able to induce apoptosis in PK-15 cells, an event that occurs at the later stages of infection.

### PRV-induced autophagy in PK-15 cells

To investigate the effect of PRV infection on the level of autophagy, the status of the proteins LC3 and SQSTM1/P62 was analyzed in PRV-infected PK-15 cells by Western blot. The expression of LC3-II was determined up to 36 hpi (MOI = 1), and the autophagy level is represented as the ratio of LC3-II/ACTB band intensities. As shown in Figure 1D, the expression of LC3-II was significantly increased at 6 hpi (*P* < 0.01). A reduced expression of LC3-II was detected from 12 to 36 hpi in PRV-infected cells compared to that at 6 hpi. These results indicate that PRV-induced autophagy occurs at the early stage of infection in PK-15 cells. Of note, the level of the autophagy marker protein LC3-II decreased progressively along with an increased expression of PRV gE protein during the course of infection time, suggesting that PRV inhibits the level of autophagy at the late stage of infection. To visualize LC3 II associated with the autophagic membrane, LC3 fused with a green fluorescent protein (GFP) was used. The LC3 can redistribute from a diffuse cellar localization to distinctive puncta cytoplasmic pattern during autophagy, revealing the recruitment of LC3 to autophagic vesicles (13). PK-15 cells were infected with PRV at an MOI of 1 following transfection with GFP-LC3 for 24 h and then observed with confocal microscopy at different time points after infection. GFP-LC3 proteins were distributed as foci in most of the PRV-infected PK-15 cells at 4 hpi. In contrast, GFP-LC3 remained diffusely distributed throughout the cytoplasm at 24 hpi both in uninfected cells and in PRV-infected cells (Figure 1E). The number of autophagic cells was quantified as the percentage of GFP-LC3 puncta expressing cells among total GFP-expressing cells. Approximately 32.4 and 14.8% of PRV-infected PK-15 cells had formed GFP-LC3 puncta at 4 and 24 hpi, respectively (Figure 1E). These results provide strong evidence that PRV infection induced autophagy at the early infection stage. In order to determine the autophagic degradation during PRV infection, P62, previously described as autophagosome cargo was analyzed using Western blot. The result showed that the level of P62 decreased at the early stage of infection and then increased at 12 hpi and later time points. Chloroquine (CQ) was used to analyze the turnover of autophagosomes. The PRV-infected and mock-infected cells were pre-treated with CQ for 4 h. The expression of LC3-II and P62 was analyzed and quantified at 2 and 8 hpi. Our results showed that CQ increased the level of LC3-II and P62 in PRV-infected cells when compared to PRV-infected cells in the absence of CQ treatment. These results demonstrate that PRV infection enhances the autophagy flux (Figure 1F). Taken together, our results indicate that PRV infection has a dual effect on autophagy: PRV enhances autophagic degradation as well as the autophagy flux at the early stage of infection and decreased autophagy level at the late stage of infection.

### Inhibition of Autophagy increases PRV-induced apoptosis

**Figure 2:**
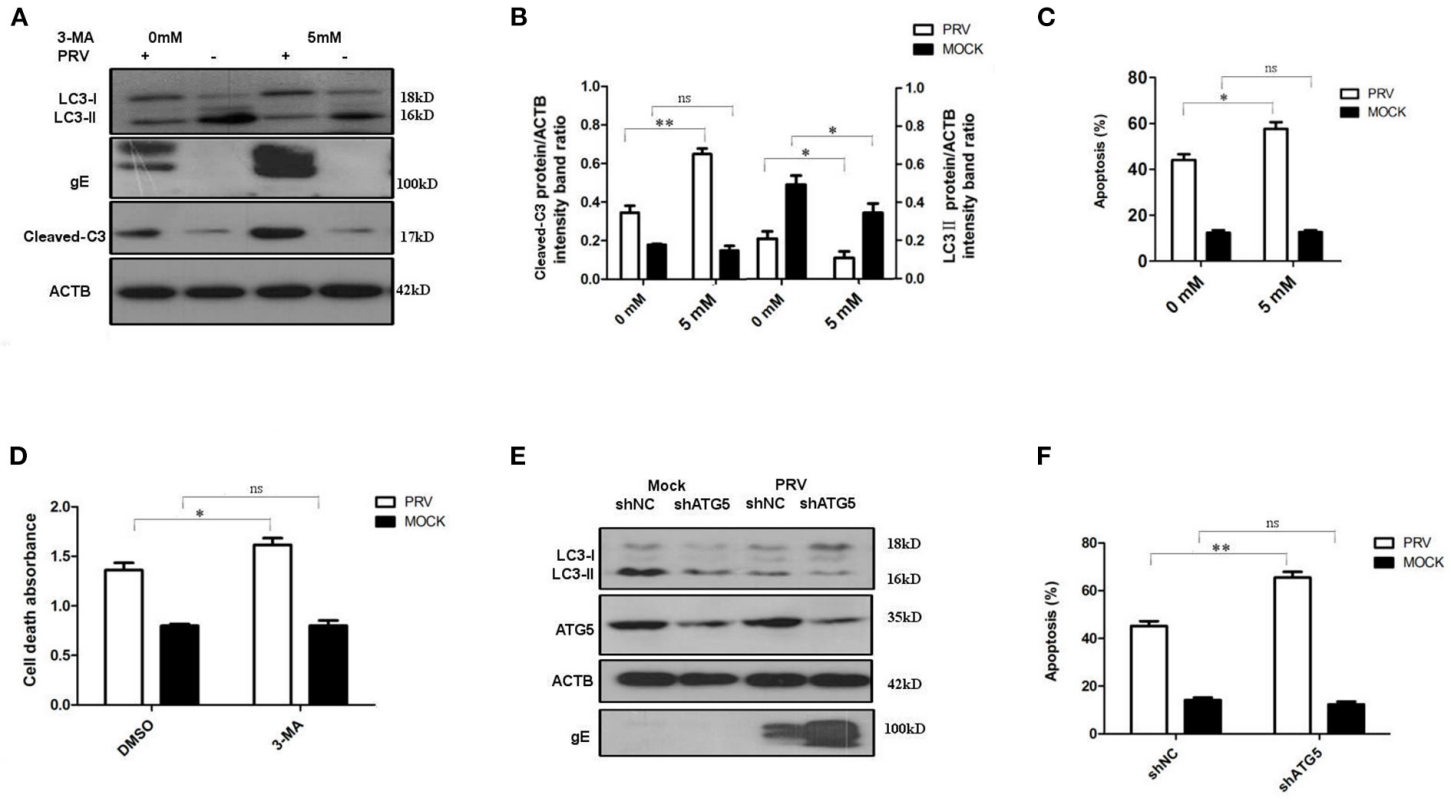
The inhibition of autophagy increases PRV-induced apoptosis. **(A)** PK-15 cells were treated with 3-MA, followed by PRV infection. The cells were lysed and analyzed by Western blot. And the statistical analysis of cleaved-caspase-3/ACTB was performed and shown in **(B)**. **(C)** PK-15 cells were treated as in **(A)** and analyzed by flow cytometry. The percentage of apoptotic cells was represented as the histogram. **(D)** The cells were treated as in **(A)** and lysed, followed by cell-death ELISA analysis. **(E)** PK-15 cells were transfected with shRNAs for 24 h, followed by PRV infection. The expression of the ATG5 protein and autophagic level was detected by Western blot. **(F)** The cells were treated as in **(E)** and analyzed by flow cytometry. The percentage of apoptotic cells was shown in the histogram. All the dates are representative of three independent experiments. The data represent the mean ± SD, and analyzed by two-way ANOVA, *P < 0.05; **P < 0.01.

The PK-15 cells were pre-treated with 5 mM 3-MA to inhibit the autophagy levels for 4 h and then infected by PRV. The effects of PRV infection on the regulation of autophagy and apoptosis response were analyzed by Western blot (Figure 2A) and flow cytometric analysis (Figure 2C) at 12 hpi. As shown in Figures 2A, B, PRV infection showed a decreased LC3-II level and enhanced cleaved caspase-3 in 3-MA pre-treated cells when compared to non-3-MA treatment cells. The flow cytometric analysis showed in 3-MA pre-treated cells an increase of apoptosis to 59% in PRV-infected cells (*P* < 0.05) (Figure 2C). The change in the rate of cell death was also analyzed by ELISA, and the results confirmed that 3-MA pretreatment significantly increases the rate of cell death in PRV-infected cells (*P* < 0.05) (Figure 2D). Next, short hairpin RNA (shRNA) targeting ATG5 was used to decrease autophagy level in PK-15 cells. The regulation of autophagy and interference efficiency is shown in Figure 2E. Treatment with shATG5 reduced the expression of ATG5 and significantly inhibited autophagy in mock- and PRV-infected cells. The Western blot results indicate that the shRNA-induced decrease of the autophagy level is paralleled by an enhanced expression of viral gE protein (Figure 2E). Subsequently, we determined the apoptosis rate in PRV-infected ATG5 knockdown cells. The flow cytometry results revealed that knockdown of autophagy with shRNA targeting endogenous ATG5 resulted in an increase in the percentage of apoptosis cells during PRV infection (Figure 2F). These results suggest that inhibition of the level of autophagy at the early stage of PRV infection may up-regulate PRV-induced apoptosis and facilitate virus replication.

### Increased autophagy level decreases PRV-induced apoptosis

**Figure 3:**
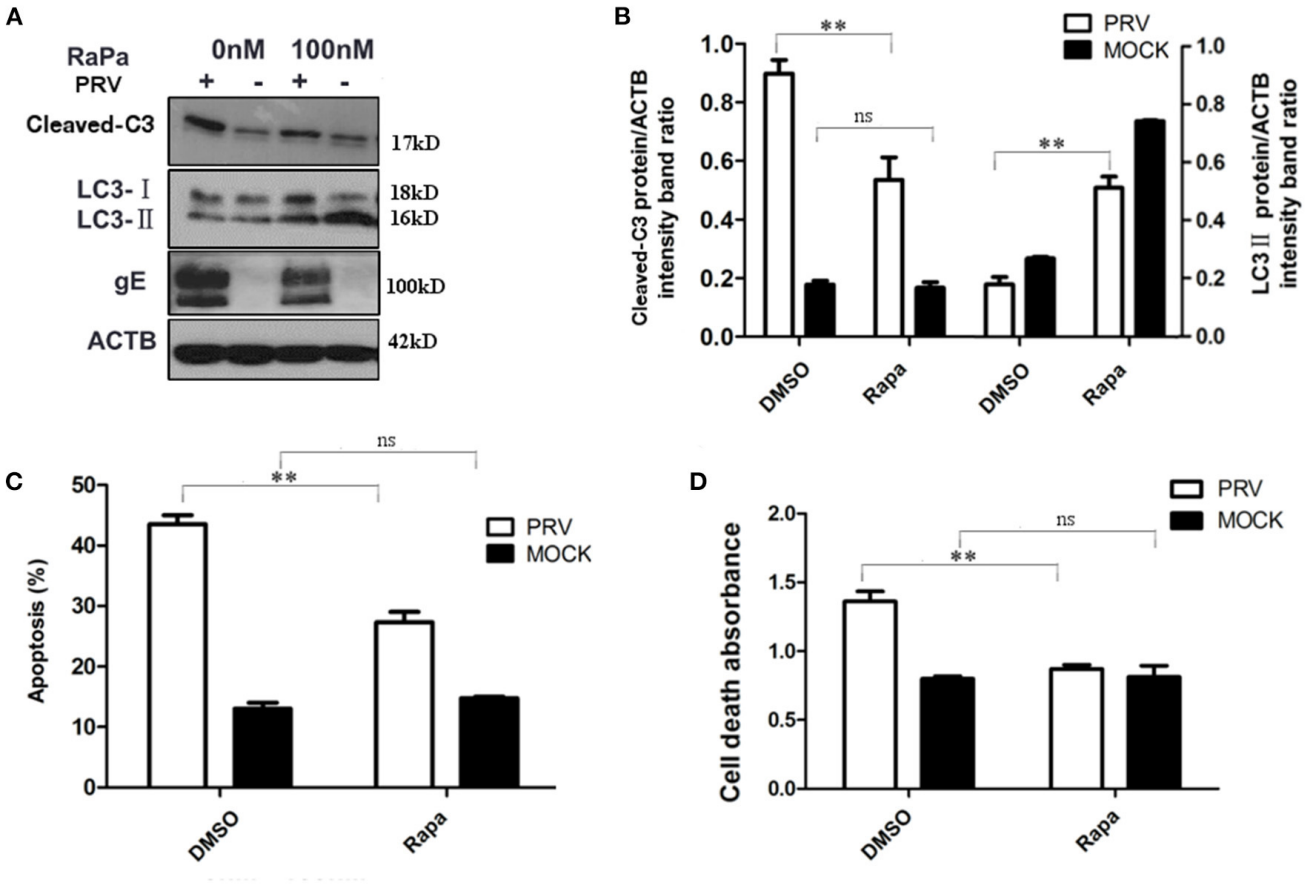
The increased autophagy decreased PRV-induced apoptosis. **(A)** PK-15 cells were treated with rapamycin, followed by PRV infection. The cells were lysed and analyzed by Western blot, and the statistical analysis of the cleaved-caspase-3/ACTB was performed, shown in **(B)**. **(C)** The cells were treated as in **(A)**, and the percentage of apoptosis cells was detected by flow cytometry. **(D)** The cells were treated as in **(A)** and lysed, and cell-death ELISA was performed. All results are representative of three independent experiments. The data represent the mean ± SD of three independent experiments and are analyzed by two-way ANOVA, **P < 0.01.

To further verify the regulation of autophagy and apoptosis at the late stage of PRV infection, the virus-induced apoptosis response was analyzed when cells were treated with rapamycin, an autophagy inducer. At 36 hpi, the cells were collected and analyzed by Western blot and flow cytometry to detect the levels of autophagy and apoptosis. The results showed that infection of PRV in 100 nM rapamycin-pretreated PK-15 cells increased the amount of LC3-II and decreased cleaved caspase-3 when compared with PRV infection of cells that had not been pre-treated (Figures 3A, B). This indicates that by enhancing the autophagy level, rapamycin compromises the effect of PRV infection, inhibiting LC3-II production at 36 hpi. Besides, the expression of PRV gE protein was notably reduced in PRV-infected rapamycin-pretreated PK-15 cells. Then we analyzed the apoptosis rate in PRV-infected rapamycin-pretreated cells by flow cytometry and ELISA. These results were consistent with the results obtained by Western blot, i.e., enhancement of the autophagy level significantly decreased the number of apoptotic cells among PRV-infected cells (*P* < 0.01) as well as the amount of the dead cells determined by ELISA (*P* < 0.01) (Figures 3C, D). Taken together, our results indicate that enhancement of the autophagy level interferes with the induction of apoptosis during PRV infection.

### Inhibition of apoptosis increases the autophagy level in PRV-infected PK-15 cells

**Figure 4:**
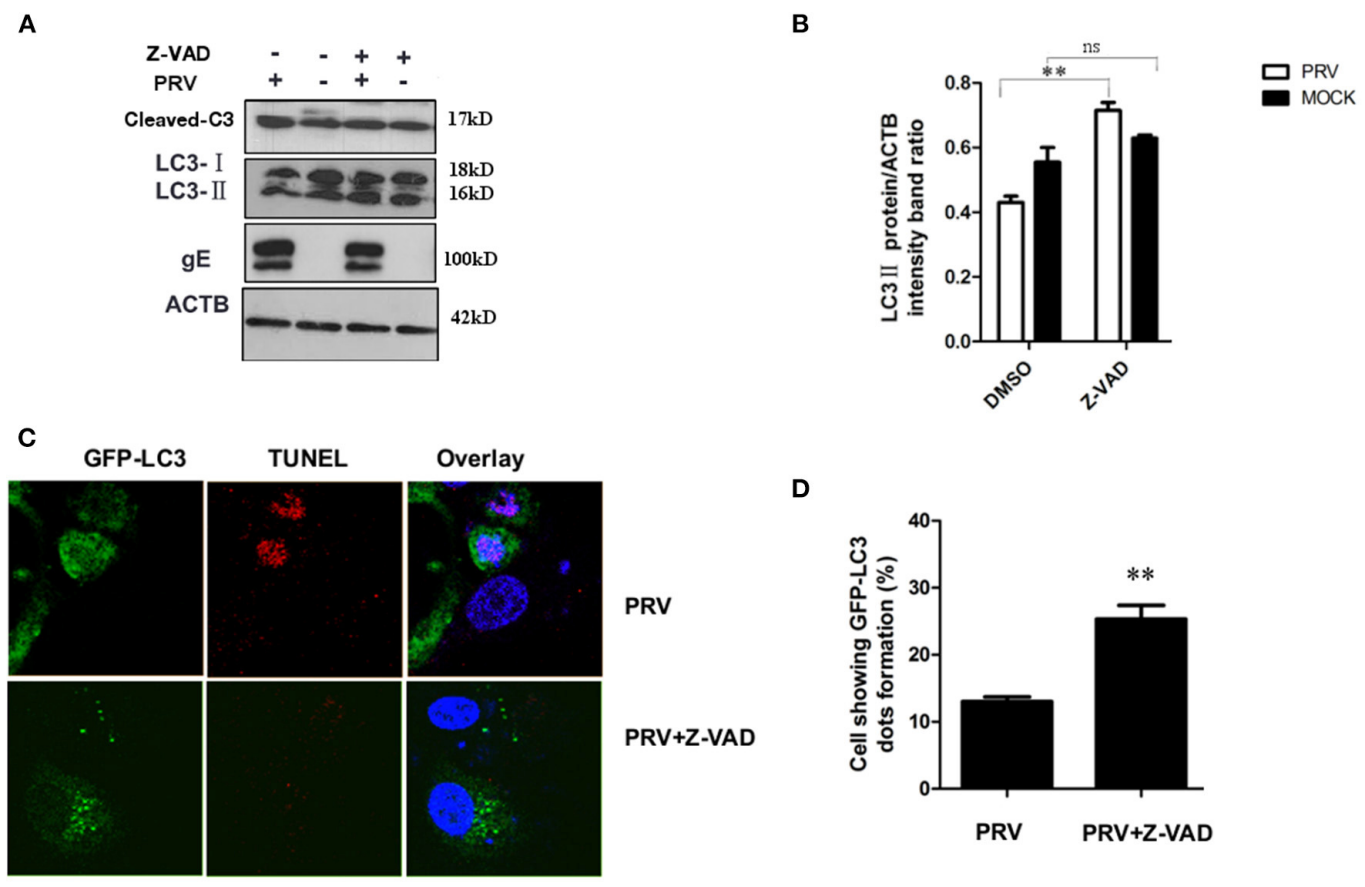
Inhibition of apoptosis increases the autophagy level in PRV-infected cells. **(A)** PK-15 cells were pre-treated with Z-VAD-FMK for 4 h, followed by PRV infection for an additional 12 hpi. The cells were lysed and analyzed by Western blot, and the statistical analysis of LC3-II to ACTB and P62 to ACTB was performed, shown in **(B)**. **(C)** PK-15 cells were transfected with the GFP-LC3 for 24 h and pre-treated with Z-VAD-FMK for 4 h followed by PRV infection. Confocal microscopy was used to locate the GFP-LC3 puncta and TUNEL stain. **(D)** the percentage of cells showing GFP-LC3 dots formation and at least 50 cells were included in each group. All the results are representatives of the three independent experiments. The data represent the mean ± SD of three independent experiments and are analyzed by one-way ANOVA or two-way ANOVA, **P < 0.01.

It has been reported that apoptosis could regulate autophagy response through caspase or other proteins (25, 26). In order to test the role of apoptosis in regulating autophagy during the late stage of PRV infection, Z-VAD-FMK (30 μM) was used to block caspase-dependent apoptosis. The expression of LC3-II and the cleaved caspase-3 after Z-VAD-FMK treatment were detected by Western blot. Our results showed that Z-VAD-FMK slightly decreased the amount of cleaved caspase-3 in PRV-infected cells. In addition, an increased expression of LC3-II was detected along with the inhibition of apoptosis by Z-VAD-FMK in PRV-infected cells (Figures 4A, B). The localization of LC3 in Z-VAD-FMK-treated PK-15 cells was visualized using GFP-LC3. Cells were treated with Z-VAD-FMK and infected with PRV followed by transfection of GFP-LC3 for 24 h and apoptotic cells were stained with TUNEL. Figure 4C showed that PRV infection induced positive TUNEL staining and diffuse GFP-LC3 distribution at 24 hpi; Z-VAD-FMK treatment decreased the number of apoptotic cells, and GFP-LC3 puncta that is a marker of autophagic vesicle formation were formed in non-apoptotic cells which showed no TUNEL stains. And the number of autophagic cells was quantified as the percentage of GFP-LC3 puncta-positive cells to total GFP-expressing cells in PRV infection and Z-VAD-FMK-treated PRV infection cells. The percentage of GFP-LC3 puncta-positive cells increased from approximately 12.4–26.7% when PRV infect on Z-VAD-FMK pre-treated PK-15 cells (Figure 4D). Our results indicate that apoptosis inhibition can increase autophagy may mediate by caspase.

### Autophagy had a more important effect on PRV replication

**Figure 5:**
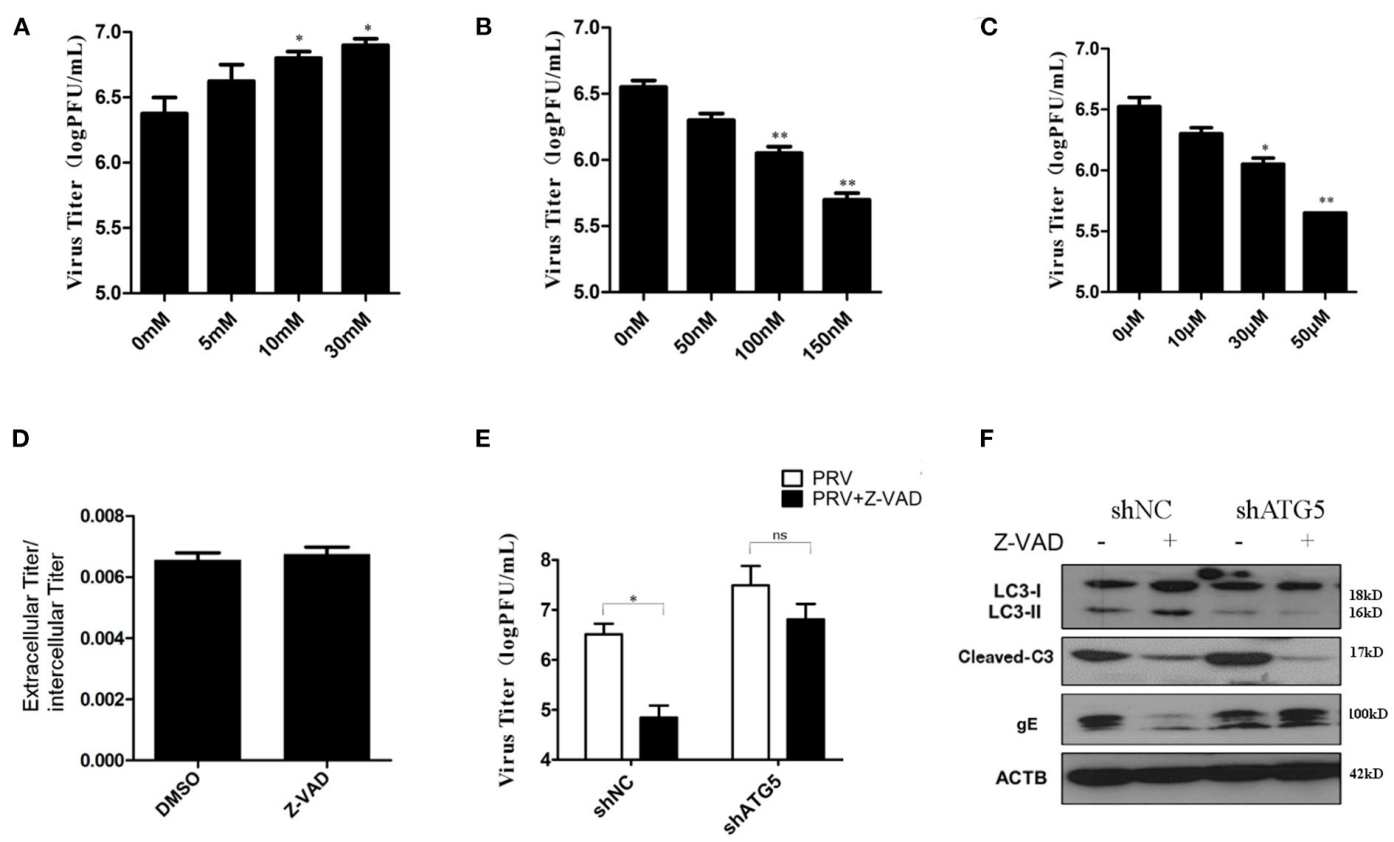
Autophagy had a more important effect in PRV replication. **(A)** PK-15 cells were treated with 3-MA followed by PRV infection (MOI = 1). The viral titer was determined by PFU assay. **(B)** PK-15 cells were treated with rapamycin, followed by PRV infection (MOI = 1). The viral titer was determined by PFU assay. **(C)** PK-15 cells were treated with Z-VAD-FMK followed by PRV infection (MOI = 1). The viral titer was determined by PFU assay. **(D)** The ratio of the virus titers in the supernatants and cell lysates was determined according to the results of the PFU assay. **(E)** The combined effect of autophagy and apoptosis on PRV infection. PK-15 cells were transfected with shATG5 for 24 h and then treated with Z-VAD-FMK for an additional 4 h followed by PRV infection. The viral titer was determined by PFU assay, and the autophagy level and apoptosis level were tested by Western blot shown in **(F)**. The data represent the mean ± SD of three independent experiments and are analyzed by two-way ANOVA, *P < 0.05; **P < 0.01.

Both autophagy and apoptosis play a role in viral infection and propagation, and our results suggest that PRV infection regulates these two pathways. To evaluate which process affected viral propagation, the amount of infectious virus in autophagy and/or apoptosis-impaired cells was measured. The autophagy inhibitors, 3-MA and shATG5, increase PRV gE protein expression (as shown in Figures 2A, D). The effect of 3-MA on virus production was analyzed by determining the infectivity (PFU assay). Concentrations of 10 and 30 mM 3-MA increased the infectious PRV in PK-15 cells (Figure 5A), which is consistent with the viral gE protein expression. Next, the effect of rapamycin on PRV replication was determined. Figure 5B showed that when cells were pre-treated with rapamycin, the virus titer was significantly reduced in a dose-dependent manner. The effect of the apoptosis inhibitor Z-VAD-FMK was determined in the same way. The amount of infectious PRV was reduced significantly (Figure 5C) when PK-15 cells were pre-treated with 10, 30, or 50 μM Z-VAD-FMK, respectively. To verify whether the Z-VAD-FMK-induced decrease of the virus titer affects virus release, the virus was collected from the supernatants as well as from cell lysates. The results show that Z-VAD-FMK treatment had no influence on the ratio of PRV titer between supernatants and cell lysates compared to that determined with control cells (Figure 5D), indicating that the apoptosis-induced increase of viral production is not due to an enhanced PRV release.

Drugs that influence either autophagy or apoptosis play roles in the replication of the virus, and autophagy and apoptosis cross-inhibit each other in PRV-infected PK-15 cells. To further verify the interaction of these two processes during PRV replication, autophagy-knockdown cells were pre-treated with Z-VAD-FMK, and the amount of infectious PRV in infected cells was assessed. As shown in Figure 5E, in shNC cells, Z-VAD-FMK treatment decreased the PRV titers, while in autophagy-impaired cells (shAtg5 cells), the Z-VAD-FMK treatment showed little or only a reduced effect on the replication of PRV, which indicates that autophagy may have a more important role in PRV replication when compared with apoptosis. The autophagy and apoptosis levels in these drug-treated cells were also determined by Western blot, as shown in Figure 5F. Based on these data, we conclude that PRV infection can be regulated by apoptosis, which acts mainly *via* inhibiting autophagy. An increased autophagy level reduces the replication of PRV in the Z-VAD-FMK-treated cells.

### ROS expression mediates PRV-induced apoptosis in autophagy-suppressed cells

**Figure 6:**
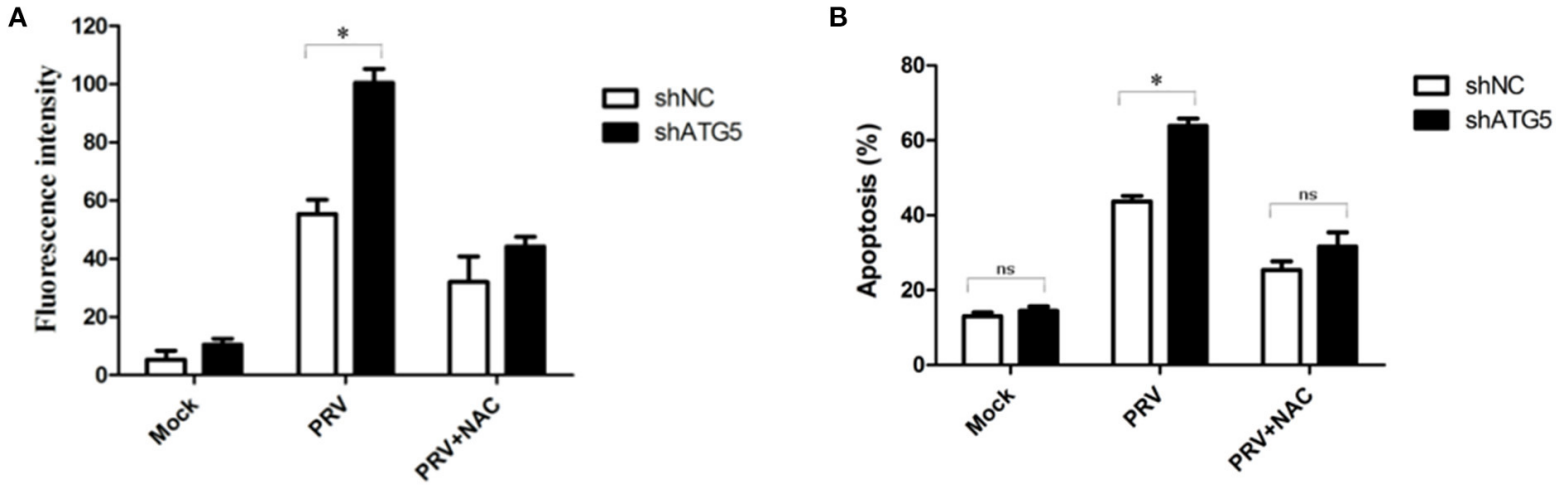
ROS expression mediates PRV-induced apoptosis in autophagy-suppressed cells. **(A)** PK-15 cells were transfected with shATG5 and shNC for 24 h and then infected with PRV. NAC was added at the infection time to eliminate ROS. ROS level was measured with DCFH-DA as previously described. The level of ROS was tested by flow cytometry. **(B)** The cells were treated as **(A)**, and then analyzed by flow cytometry to detect the levels of apoptosis. The data represent the mean ± SD of three independent experiments and are analyzed by two-way ANOVA, *P < 0.05.

It has been reported that a high level of ROS is a primary cause of apoptosis under both physiologic and pathologic conditions, and the oxidized substance is degraded during the autophagy process. Therefore, we analyzed whether ROS are involved in the cross-talk between PRV-induced apoptosis and autophagy. For this purpose, we determined the intracellular ROS levels and the apoptotic rates in autophagy-knockdown cells (ATG5 shRNA) by flow cytometry, as shown in Figures 6A, B. The addition of the ROS inhibitor (NAC) at a concentration of 50 mM abolished the staining confirming the specificity of this assay. The ROS level in shATG5-treated cells was significantly higher than they were in shNC-treated cells at 48 hpi (Figure 6A). The percentage of apoptosis in PRV-infected shATG5 knockdown cells was about 63% which was notably higher than that in virus-infected shNC cells, approximately 42%. Furthermore, the NAC treatment decreased the percentage of apoptotic cells in PRV-infected autophagy-impaired cells. However, no different apoptosis level was detected between PRV-infected shATG5 and shNC cells in the presence of NAC (Figure 6B). These data suggest that ROS accumulation mediates the apoptosis in autophagy-impaired cells.

## Discussion

Different forms of cellular stress may be triggered when cells are suffering from a pathogen infection. Autophagy, apoptosis, and their tight interaction play important roles in viral infections and determine the fate of the cell. Autophagy and apoptosis, as two important cellular responses against virus infection, are involved in the establishment of PRV latency and reactivation. The appearance of these two responses is not synchronous during PRV infection, and the underlying interactions between them have not been well-characterized. For a coronavirus and a paramyxovirus, it has been suggested that preventing apoptosis at the early stage of infection contributes to viral replication and results in a higher yield of virus (27, 28). We found that inhibition of apoptosis decreased the replication efficiency of PRV in PK-15 cells and resulted in an up-regulation of the autophagy level. These results are consistent with previous studies that autophagy or autophagy genes may play antiviral roles (29). Besides, inhibition of autophagy at the early stage of PRV infection can up-regulate virus-induced apoptosis and facilitate PRV replication, which means autophagy can interfere with the apoptosis process and control PRV infection. Therefore, PRV infection induces a switch from autophagy to apoptosis to facilitate viral progeny production, which closely depends on the interconnection between autophagy and apoptosis.

In previous studies, it has been suggested that either autophagy or apoptosis can be induced by PRV infection (30, 31). Still, the cross-talk between these two cellular processes has been poorly investigated. Therefore, a comprehensive analysis of the interconnectivity of autophagy and apoptosis is essential for understanding PRV infection *in vitro*. We confirmed that PRV induced autophagy at the early stage of viral infection and caused apoptosis in a later stage, which indicates that autophagy was triggered earlier than apoptosis in PRV-infected cells. Interestingly, we found that during the PRV infection in PK-15 cells, autophagy and apoptosis affected each other mutually. PRV-induced apoptosis was increased when autophagy level was inhibited, and enhanced autophagy levels decreased the occurrence of apoptosis. Therefore, our study indicates that PRV-induced autophagy and apoptosis influence each other and affect viral replication. Thus, studies on PRV-induced autophagy and apoptosis should not be separated as two different aspects but considered as a continuum. Because the molecules used to regulate autophagy and apoptosis are involved in a variety of cellular signaling processes, the effect on viral replication may also cause by other mechanisms, such as rapamycin-mediated mTORC1 inhibition, not only activates autophagy, and also lead to diminished phosphorylation of 4E-BP1 and inhibition of some transcript expressions.

Several mechanisms may account for the capacity of autophagy to limit apoptotic cell death. Reactive oxygen species (ROS), as highly reactive molecules, are generated as by-products of cellular metabolism and are associated with various cellular processes (32). On the one hand, oxidative stress can promote autophagy formation, but on the other hand, autophagy can degrade the oxidized substances to reduce oxidative damage (33). A previous study has shown that PRV infection can induce apoptosis *via* oxidative stress signaling in PK-15 cells (31), while increasing intracellular ROS levels, which causes an autophagy and apoptosis response. We confirmed that PRV infection caused significant ROS production and induced a high level of apoptosis in autophagy-impaired cells. This is consistent with autophagy, which is considered an important mediator of ROS production to protect the cells from apoptosis (34), and the inhibition of autophagy causes massive accumulation of oxidative stress, leading to the process of apoptosis (35). Therefore, ROS may participate in the cross-talk between autophagy and apoptosis during PRV infection.

A previous study has shown that PRV infection is able to activate autophagy even before viral genome replication (30), and we further found that autophagy can be detected as early as 2 hpi in PK-15 cells, while apoptosis was shown 12 hpi, which is the crucial time point for the switch from autophagy to apoptosis. It has been reported that the expression of US3 inhibits autophagy during PRV replication, and overexpression of US3 may inhibit apoptosis (10, 30). Therefore, we speculated that the viral genome activated the autophagy process before PRV genome translation. But the expression of US3 inhibits autophagy, and a low level of autophagy causes the accumulation of ROS (35). As a consequence, massive ROS triggered an apoptosis process. Although, it has been reported that transient expressing US3 protein can suppress apoptosis (9, 10). Such overexpression of US3 protein may occur in the late stage of virus infection, i.e., at 12 hpi and later. The effects of US3 on mediating autophagy and apoptosis are only one aspect of the complex stress response during PRV infection.

Apart from viral proteins, many cellular factors that can cause cell death also have an influence on autophagy and apoptosis cross-talk. It has been previously reported that activation of apoptosis-associated caspase may result in the cleavage of various essential pro-autophagic proteins and thus shut down the autophagy response (25). Our data show that the autophagy level was enhanced when PRV infection occurred in caspase-impaired cells indicating that the low amounts of caspase protease are presumably not sufficient to degrade the pro-autophagic proteins efficiently and trigger autophagy finally, which suggests that the caspase might play a role in the switch from apoptosis to autophagy during PRV infection, and this mechanism may accelerate the apoptosis, in the final stage of viral infection (15).

In conclusion, we have shown that autophagy activated at the early stage of PRV infection inhibits apoptosis and that apoptosis inhibits autophagy at the last step. We speculate that the decreased autophagy facilitates viral replication. Our studies provide possible molecular mechanisms for a cross-talk between apoptosis and autophagy induced by PRV infection in porcine cells. This suggests that these cell death processes should be considered as a continuum rather than an entirely separate process. Our findings provide a novel insight into this virus-host interaction, which may help to exploit apoptosis and autophagy pathways as novel targets for antiviral therapy.

## Data availability statement

The original contributions presented in the study are included in the article/Supplementary material, further inquiries can be directed to the corresponding authors.

## Author contributions

MS and XC designed and conceived the experiments. MS, LH, HS, Y-dT, and CL performed the experiments. YL and SW contributed reagents and materials. LH and Y-dT analyzed the data, and interpreted the conclusions. MS and FM wrote and reviewed the manuscript. XC and FM entirely supervised the project. LQ prepared the revised manuscript. All authors contributed to the article and approved the submitted version.
