# Supplementary material for: The relationship between autophagy and apoptosis during pseudorabies virus infection
Source: Front Vet Sci. 2022 Dec 20;9:1064433. doi: 10.3389/fvets.2022.1064433 (PMC9810027; doi:10.3389/fvets.2022.1064433)
Supplement: Supplementary file 1 [file Table_1.DOCX]

Table S1. The sequences used for shRNA plasmids construction

| Gene | Target sequence (5’- 3’) |
| --- | --- |
| shATG5 | GCAACTCTGGATGGGATTG |
| ShNC | GAATCTGATGGACTTGGTC |
